# Early crack detection using modified spectral clustering method assisted with FE analysis for distress anticipation in cement-based composites

**DOI:** 10.1038/s41598-021-99010-8

**Published:** 2021-10-04

**Authors:** Ajitanshu Vedrtnam, Santosh Kumar, Gonzalo Barluenga, Shashikant Chaturvedi

**Affiliations:** 1 Department of Mechanical Engineering, Invertis University, Bareilly, UP 243001 India; 2 Departamento de Arquitectura, Escuela de Arquitectura -Universidad de Alcalá, Alcala de Henares, Madrid, Spain; 3 Department of Electronics and Communication Engineering, Invertis University, Bareilly, UP 243001 India

**Keywords:** Engineering, Mathematics and computing

## Abstract

The present work reports an efficient way of capturing real-time crack propagation in concrete structures. The modified spectral analysis based algorithm and finite element modeling (FEM) were utilised for crack detection and quantitative analysis of crack propagation. Crack propagation was captured in cement-based composite (CBC) containing saw dust and M20 grade concrete under compressive loading using a simple and inexpensive 8-megapixel mobile phone camera. The randomly selected images showing crack initiation and propagation in CBCs demonstrated the crack capturing capability of developed algorithm. A measure of oriented energy was provided at crack edges to develop a similarity spatial relationship among the pairwise pixels. FE modelling was used for distress anticipation, by analysing stresses during the compressive test in constituents of CBCs. FE modeling jointly with the developed algorithm, can provide real-time inputs from the crack-prone areas and useful in early crack detection of concrete structures for preventive support and management.

## Introduction

Cracks and distress in concrete structures are generally due to restrained shrinkage, improper load balancing or material degradation. Their early detection and repair is a priority for proper maintenance as their development might lead to fatal damage and structural collapse. Before image processing techniques were developed, man-made inspection was the only crack inspection technique, but it is dreggy and requires considerable time dedication and skilled staff^1,2^. Image processing is cheap, accurate, and can be automated, becoming the alternative of this manual approach for structural inspection.

Image processing methods can provide a highly precise crack detection results based on local features of the crack image^3^. There are many powerful techniques in image processing for crack detection and takes a different form of images captured through the digital camera, Infra-red (IR) camera, ultrasonic imaging, laser imaging, time of flight diffraction (TOFD) and various other imaging technologies. The generalised process of crack detection using image processing involves image pre-processing and then utilise the preprocessed picture for the feature extraction and parameter estimation required for the determination of crack length and direction of propagation^4^.

The images captured using a digital camera for concrete crack detection is frequently reported^5–7^. These studies have attempted to explain the role of camera imaging for crack detection through threshold segmentation, neural network, image stitching, and acoustic emission. The crack detection using the structural feature (morphological and multidirectional shape of the crack) based algorithm on camera images has been developed and tested in a few studies^8,9^. Different filters based on region, edge and contrast features of the crack images have been used to find cracks in the multi-step model^10–13^. Some authors developed an artificial intelligence-based depth analysis model for the crack depth calculation^6,14–16^.

However, a cost-effective, reliable commercial application of the image processing technologies for crack detection in the structures still requires significant research efforts. Many researchers are working in this direction; Fernandez et al.^1^ proposed a method to minimise the cost of crack repair by developing an early crack detection system utilising logarithmic transformation, bilateral filter, canny, and morphological filter. Oliveira et al.^2^ proposed a fully integrated system based on unsupervised learning for automatic detection and characterisation of cracks, which doesn’t require the manual labeling of the samples. Wavelet-based crack image decomposition into different frequency sub-band was demonstrated by Zhou et al.^17^ for the detection of cracks in a different direction. Zhong et al. used an ultra-efficient crack detection algorithm with a second percolation model to address the issue of unclear and tiny cracks to be detected effectively^18^. Many other authors have raised the issue of detection of non-crack parts such as image shadow and stains, which alternatively affect the accuracy of the system and solve the problem by using various types of noise removal filters^19,20^. Hoang et al.^21^ demonstrated a program for surface crack investigation using Min–Max Gray Level Discrimination by adjusting the gray intensity for accurate crack detection. Sheerin et al.^22^ reported a study of Otsu’s based thresholding method for the classification of the different types of cracks. Arun et al.^23^ presented an analysis based on multiple features for the measurement of length, width, and direction of propagation of the crack. Kaur et al.^24^ reported a comparative study for extracting curves, edges, and other features of the crack and pointed out that no single method is sufficient for every image type.

Compared to other methods available for image segmentation, thresholding is the simplest and fastest amongst all. Pal et al.^25^ studied the impact of thresholding and its associated methods such as hidden Markov random field (HMRF), Markov Random Field, and K-means Clustering for accurate crack detection. Clustering is a powerful technique^26^, and Sathya et al.^27^ discussed some important clustering methods such as k-means, improved k means, fuzzy c-mean (FCM), and improved fuzzy c-mean algorithm (IFCM) to determine the even small crack in the concrete structure. Jorden et al.^28^ presented two algorithms, one for spectral clustering and another for similarity matrix, to derive a new cost function for spectral clustering based on error measurement.

Minimising this cost function concerning the partition leads to a new spectral clustering algorithm. In this way, Ng et al.^29^ reported modifications in spectral clustering as the clustering method has unresolved issues related to the eigenvalues and eigenvectors. Luxburg et al.^30^ investigated the consistency of spectral clustering by analysing the convergence of eigenvectors of the normalised and un-normalised Laplacian matrices on random samples under standard assumptions. Tung et al.^31^ suggested a modified form of spectral clustering, which includes a combination of block-wise processing and stochastic ensemble consensus for solving the most common challenge faced in image segmentation methods based on spectral clustering viz. scalability. Rohe^32^ examined spectral clustering under the more general latent space model in which the eigenvectors of the normalised graph Laplacian asymptotically converge to the eigenvectors of a “population” normalised graph Laplacian.

Noise, illumination conditions and macrotexture are some factors that can weaken crack information in captured images. Jin et al.^33^ developed a crack detection method based on spectral clustering. The proposed method worked not only on the local features (gray features) but also considered the step edge, roof, and line profile to improve the accuracy of the crack detection.

Although various methods have been proposed on crack detection using image processing, their accuracy still requires improvement, especially if the noise is present in the image acquisition environment. Thus, the first objective of the study was to develop an image processing algorithm for crack detection based on the modifications in the spectral clustering method to overcome stated challenges. However, this algorithm needs to take into account concrete composition, as it has been reported that the fracture initiation and crack propagation of concrete depend on the type of Supplementary cementitious material (SCM) incorporated^4,7–13^.

Many types of supplementary cementitious materials (SCM) have been used to produce green cement-based composites (CBCs)^34–52^, becoming encouraging alternatives for decreasing Ordinary Portland Cement consumption and volume of waste disposed on land^34^. The manufacturing process of OPC results in considerable Carbon dioxide (CO_2_) emission.

Waste wood dust is readily available as SCM at no cost in India^53,54^. Thus, the second objective of this study was to define the crack propagation pattern of CBC under compressive strength analysed combining FE quantitative analyses and SEM qualitative assessment. Finally, the effect of wood dust inclusion as SCM on the cracking pattern of a green CBC was also studied. Collectively, investigating these multidisciplinary aspects propose a novel way for unmanned inspection of the damage in the structure for appropriate maintenance.

## Materials and methods

### Sample preparation and compression testing

The concrete cube samples and green CBC samples of 150 mm × 150 mm × 150 mm were prepared for compression testing and crack propagation investigation. The amount of OPC, natural sand, aggregate (19 mm), and water were 335 kg/m^3^, 871 kg/m^3^, 1083 kg/m^3^, and 225 kg/m^3^, respectively in the concrete cubes. The water to cement ratio was 0.67. The OPC purchased from the local vendor complies with ASTM C150. The natural sand and aggregate conform to ASTM C33-16. The specific gravity, absorption, and loose density (Kg/m^3^) of natural sand and aggregate were 2.39 and 2.61, 1% and 0.39%, 2002.4 and 1818.1, respectively. The standard ASTM test procedures followed for evaluating the physical properties of the concrete. The green CBC samples included 10% of wood dust as a replacement for the OPC. The particle size of the wood dust was less than 90 µm as obtained by the sieve analysis. A total of 10 cubical samples (5 of each type) were prepared by casting in steel molds of 150 mm × 150 mm × 150 mm with the assistance of vibrator. The samples were removed from the steel molds after 24 h and cured in water for 28 days at room temperature (22–26 °C). For verification of the FE model, three additional green CBC samples were prepared; the samples were having surplus wood dust (approximately 0.01% by volume) at the upper corners of the cube incorporated manually during filling of material in steel molds. The compression testing machine (CTM) was used for evaluating the compressive strength and the SEM micrographs of fractured concrete and green CBC samples for determining the reasons for failure.

### Crack detection using modified spectral clustering

A survey of crack detection algorithms including image processing, neural network, machine and deep learning based methods is available in the literature^55^. An 8-Mega Pixel mobile phone camera has captured crack initiation and propagation in green CBC samples during the compression test. The ordinary camera contributes to the lower cost requirement for the developed system. The images of the crack developed during the compression test were captured. Further, the spectral clustering method was used to detect the crack edges. For accurate crack detection, features for differentiating the cracks from the background were selected.

In general, the gray features for crack are derived from its roof, step change, and line profile^33^. The shape, contour, and texture information along with the gray features were used for modeling a global descriptor required for high accuracy in crack detection. This allows the detection of gray features on points where sharp intensity change occurs. The pattern of orders in-phase component present at edges was studied using Fourier transform. The phase component obtained thus used for oriented energy calculation for crack edge pixels in the image.

**Figure 1:**
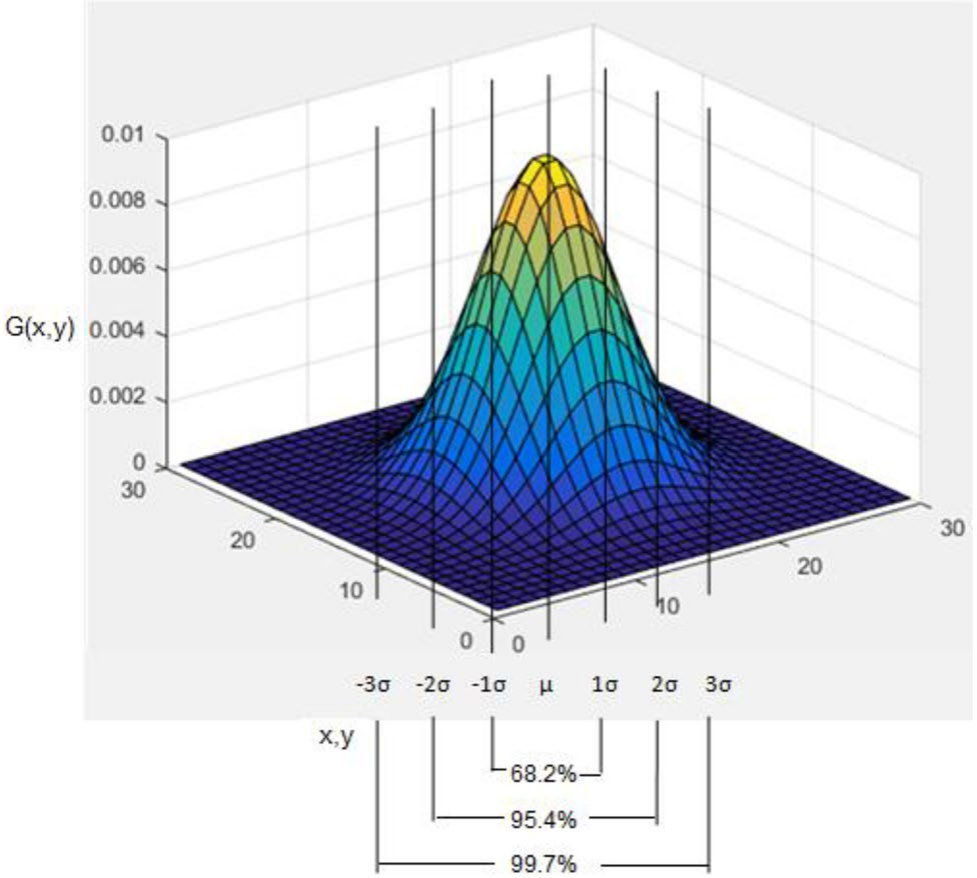
Two-dimensional Gaussian distribution surface curve.

The phase congruency is basic building block for the spectral clustering. The alternative solution of image compression is the detection of image features using the phase congruency model. This model considers that the processed image format should be high in information and low in redundancy. Therefore, rather than searching for points where there are sharp changes in intensity, the phase congruency model looks for patterns of orders based on the phase component analysis of the Fourier transform. In general, the phase congruency is calibrated through local energy oriented for edge detection. One of the major drawbacks with phase congruency based edge detection is their sensitive to the noise. Phase congruency is a clumsy parameter to assess, and this is closely associated with a standard known as oriented energy. Oriented energy is the enormity of an envelope function of the subject under measurement. The higher magnitude values in the oriented energy graphs appear at the same values in the phase congruency function. In image processing, the use of oriented energy instead of phase congruency provides independence from intensity contrast for simplification of calculation. In the present work, for reducing the sensitivity to noise present in the image, a differential phase congruency was utilised instead of phase congruency. Inclusion of differential phase has improved the accuracy of the spectral clustering. The images for concrete structure may have considerable noise, thus, this modification in spectral clustering method is useful for improving the accuracy in crack detection. Further, a pairwise comparison on pixels performed through spectral clustering to divide the pixels into two clusters. These clusters are organised by oriented energy through phase congruency associated with crack edge pixels. The differential phase congruency based method takes the signal as a gray scale image and scales it at s = 0.5 for all the test images to be used in clustering through Poisson filtering. In order to remove signal related noise especially in low intensity regions of the image Poisson filtering is performed. For an image $f\left( {x,y} \right)$ and for the phase vector $r\left( {x,y} \right)$ the differential phase vectors are calculated using Eq. (1):

1 $$ \frac{{\partial r\left( {\left( {x,y} \right),s} \right)}}{\partial s} = \left( {\theta - sin\theta cos\theta \frac{{\partial \frac{{v\left( {\left( {x,y} \right),s} \right)}}{{\left| {v(\left( {x,y} \right),s} \right|}}}}{\partial s} + \frac{{u\left( {\left( {x,y} \right),s} \right)\frac{{\partial v\left( {\left( {x,y} \right),s} \right)}}{\partial s} - v\left( {\left( {x,y} \right),s} \right)\frac{{\partial u\left( {\left( {x,y} \right),s} \right)}}{\partial s}}}{{u^{2} \left( {\left( {x,y} \right),s} \right) + \left| {v(\left( {x,y} \right),s} \right|^{2} }}} \right) $$

where, s and r (x,y) stands for scale and phase vectors; other are standard notations. The process of phase congruency to calculate the oriented energy cannot be directly implemented over an image which a 2-dimension set of discrete values (pixels). Thus, the Gaussian function (Eq. 2) was used for local energy calculation in a 2-Dimensional point spread distribution, and this was accomplished through convolution.

2 $$ {\text{G}}\left( {{\text{x}},{\text{y}}} \right) = \frac{1}{{2{{\pi \sigma }}^{2} }}{\text{e}}^{{ - \frac{{{\text{x}}^{2} + {\text{y}}^{2} }}{{2{\upsigma }^{2} }}}} $$

where, ${\upsigma }^{2}$ and G(x,y) stands for variance and Gaussian function; other are standard notations. The value of x and y are chosen concerning the 2-D image where x is the distance from the origin in the horizontal axis, y is the distance from the origin in the vertical axis, and σ is the standard deviation of the Gaussian distribution. The illustration of the Gaussian filter is shown as the integer-valued kernel in Fig. 1 with respect to every pixel value p(i, j). The values of the mask defined such that it approximates the distribution of the Gaussian with a standard deviation of 1.
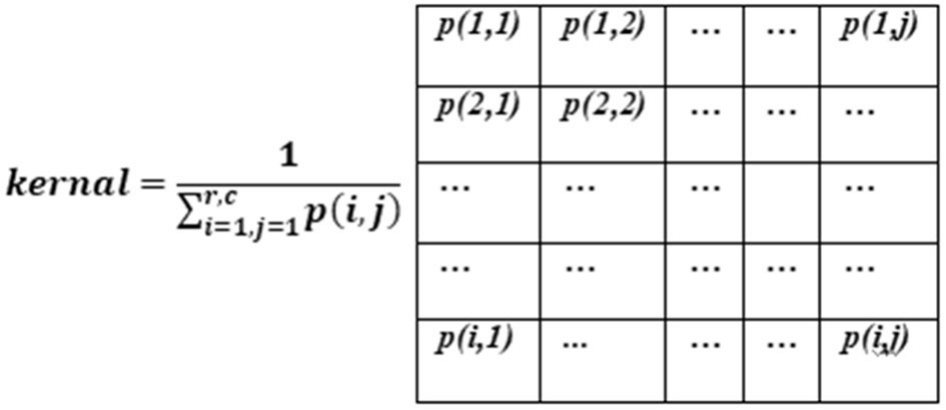

Local feature for the concrete and green CBC samples thus obtained from the Gaussian filter ${\text{ K}}_{{{\text{GF}}}} = \left( {{\text{A}} + {\text{iB}}} \right)$, where real part (A) of the complex exponential function is the second derivative of gauss function and imaginary part (B) is the Hilbert transform of the gauss function. The local energy function is obtained by convolving the complex exponential filter with crack image C_img_. Therefore, the local energy (LE) function is given by Eq. (3).

3 $$ \begin{gathered} {\text{LE}} = \left| {{\text{C}}_{{{\text{img}}}}\,*\, {\text{K}}_{{{\text{GF}}}} } \right| \hfill \\ \left| {{\text{LE}} = {\text{C}}_{{{\text{img}}}} {\,*\,}\left( {{\text{A}} + {\text{iB}}} \right)} \right| \hfill \\ {\text{LE}} = \left| {{\text{C}}_{{{\text{img}}}}\,*\, {\text{A}} + {\text{i C}}_{{{\text{img}}}}\,*\, {\text{B}})} \right| \hfill \\ \end{gathered} $$

where, LE, C_min,_ K_GF_, A and B stands for local energy, crack image, Gaussian filter, real part and imaginary part respectively. The oriented energy (${\text{OE}}_{{{{\varphi }},{\upsigma }}}$) at angle φ and neighborhood radius σ around the crack image pixel (x,y) is given by Eq. (4).

4 $$ {\text{OE}}_{{{{\varphi }},{\upsigma }}} = \left| {{\text{C}}_{{{\text{img}}}}\,*\,{\text{g}}_{1}^{{{{\varphi }},{\upsigma }}} + {\text{i C}}_{{{\text{img}}}}\,*\,{\text{g}}_{2}^{{{{\varphi }},{\upsigma }}} )} \right| $$

where, ${\text{OE}}_{{{{\varphi }},{\upsigma }}}$, φ_,_ σ, stands for oriented energy, angle, and radius respectively; other are standard notations. The ${\text{OE}}_{{{{\varphi }},{\upsigma }}}$ is maximum at the edge of the crack due to the grey features of the crack. These points have sharp information of step change, roof, and line profile for making the crack detection easier. The values of φ and σ could be adjusted for crack detection in multiple directions. The measurement of similarity makes it possible to understand how each pair of the pixel is similar or apart from the pixel at the contour that depicts the crack. However, the quantitative analysis done for the dissimilarity becomes the basis for developing a similarity or distance matrix.

**Figure 2:**
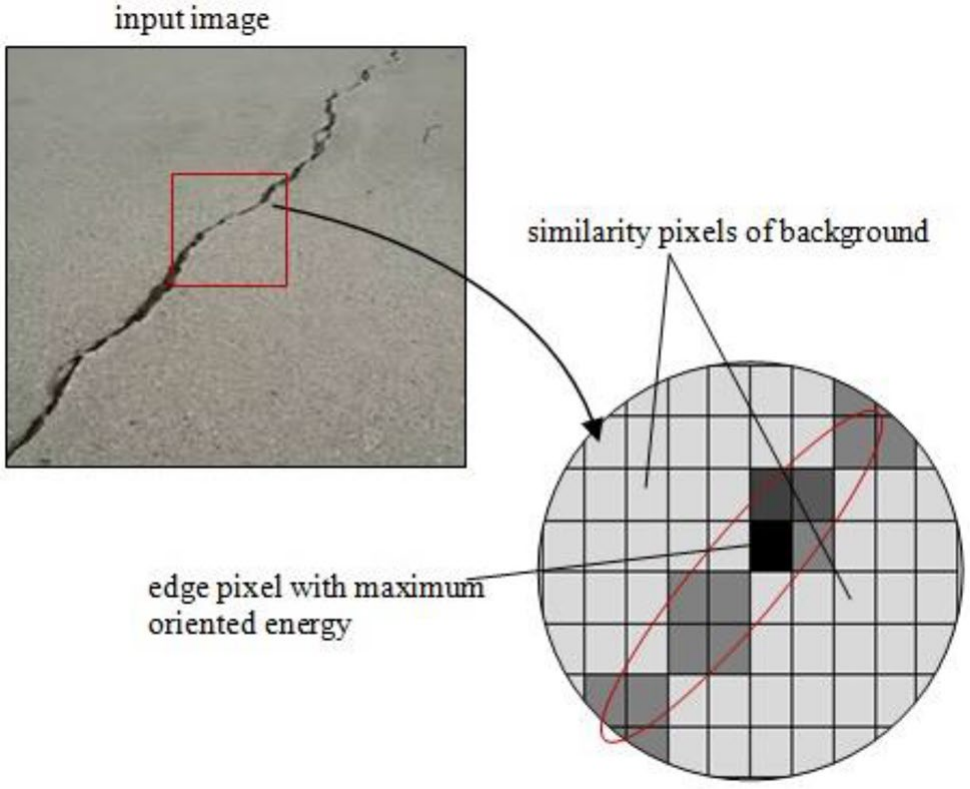
Illustration of dissimilarity between pixel p(i, j) and q(m, n).

Figure 2 gives an illustration of dissimilarity between pixel p(i,j) and q(m,n) and it accords to Eq. (5).

5 $$ D_{{{\text{edg}}}} \left( {p_{i, \, j} , \, q_{m,n} } \right) \, = {\text{ OE}}_{{{\text{con}}}} (\hat{x},\hat{y}) - {\text{avg}}\left\{ {{\text{OE}}_{{{\text{con}}}} \left( {p_{i,j} } \right) \, + {\text{ OE}}_{{{\text{con}}}} \left( {q_{i,j} } \right)} \right\} $$

The elements of the similarity matrix are calculated by Eq. (6).

6 $$ W_{{{\text{edg}}i, \, j}} = \, \exp \, \left( { - D_{{{\text{edg }}i, \, j}} } \right) $$

In Eqs. (5) and (6), the symbols stand for the standard notations. The spectral clustering is different from spatial analysis, which directly works on sample space. It is a graph theory-based method used to describe the belongingness of one object to the other. However, spectral clustering converts the spatial sample classification to the global optimal solution of graph theory. The Ncut algorithm was used as a final element of the spectral clustering to detect the crack edges. The essential terms used in Ncut method involves the assumption of graph theory such that:

1. Image is considered as a weighted graph.
2. Nodes are considered as pixels in the image and,
3. An Adjacency matrix is considered as a similarity matrix W.

The adjacency matrix as a similarity matrix has been calculated on oriented energy associated with the crack edge. The similarity matrix has the form in graph theory as:

$$ W_{i,j} = \left\{ {\begin{array}{*{20}l} {w_{i,j}:{\text{dissimilarity}}\,{\text{measure}}\,{\text{at}} \left( {i,j} \right)} \\ {0:{\text{if}}\,{\text{no}}\,{\text{relationship}}\,{\text{found}}\,{\text{at}} \left( {i,j} \right)} \\ \end{array} } \right. $$

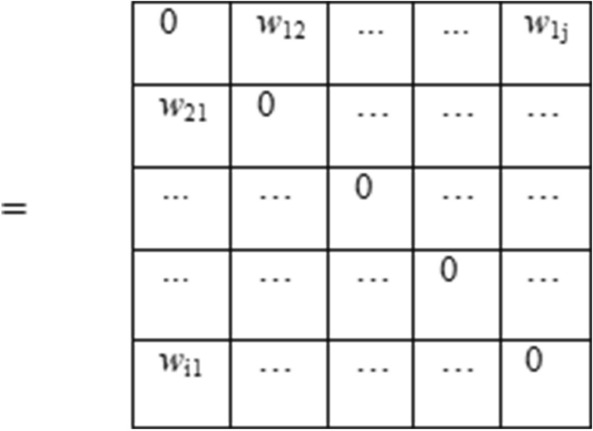


The elements of the diagonal matrix D is defined from the similarity matrix whose (i,i)_th_ elements are taken as the sum of the i_th_ row (Eq. 7).

7 $$d_{i}  = \sum _{{\{ \left. j \right|(i,j) \in E}} W_{{ij}}$$

From the definition of the Adjacency and Diagonal matrix the Laplacian matrix is then given by Eq. (8).

8 $$ L = W - D $$

Alternatively, the elements of the Laplacian matrix are defined by Eq. (9).

9 $$ L_{ij} = \left\{ {\begin{array}{*{20}l} {d_{i} }: & {{\text{if}}\,i = j} \\ { - w_{ij} }: & {{\text{if}}(i,j)\,{\text{is}}\,{\text{an}}\,{\text{edge}}} \\ 0: & {{\text{if}}\,{\text{no}}\,{\text{edge}}\,{\text{between}}(i,j)} \\ \end{array} } \right. $$

In Eqs. (7, 8 and 9), the symbols stand for the standard notations. The characteristics of the Laplacian matrix are then implemented through eigenvalues and eigenvectors. The gradient of the eigenvector was used to determine the crack edge as eigenvector contains the vital information of the crack. The coding for the algorithm was executed using MATLAB.

### Finite element model

The 3D FE based numerical model was constituted using COMSOL 5.4 for analysing stresses and deformation in concrete and green CBC during compressive loading. The representative volume method (RVM) was employed for making the model computationally inexpensive^56,57^. The geometry of the representative volume was constituted considering the volume proportion of each element for concrete and the green CBC separately.

**Figure 3:**
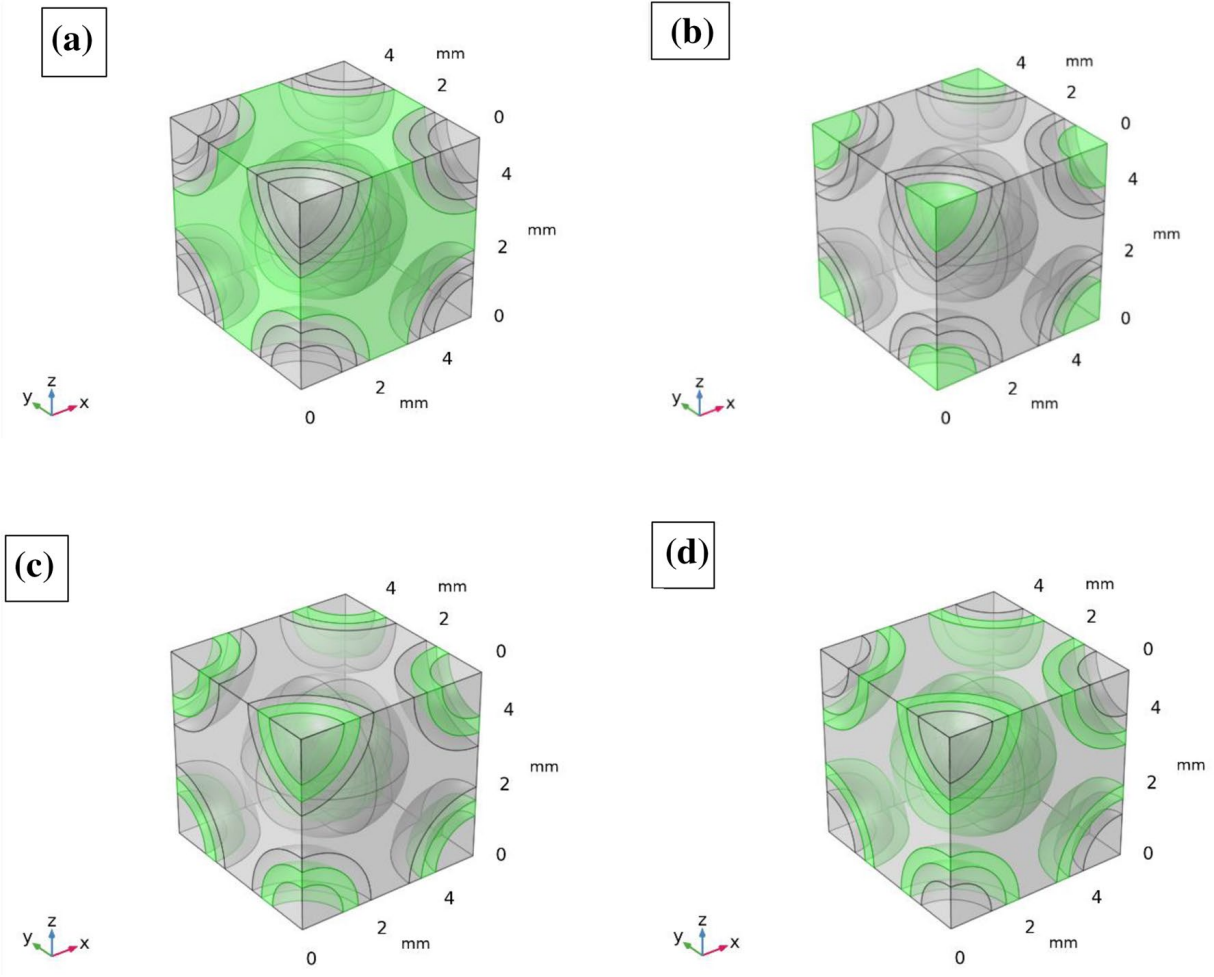
(**a**) Coarse aggregate (**b**) Fine aggregate (**c**) Cement (**d**) Wood dust zone of Green cement-based composites highlighted in green color.

**Figure 4:**
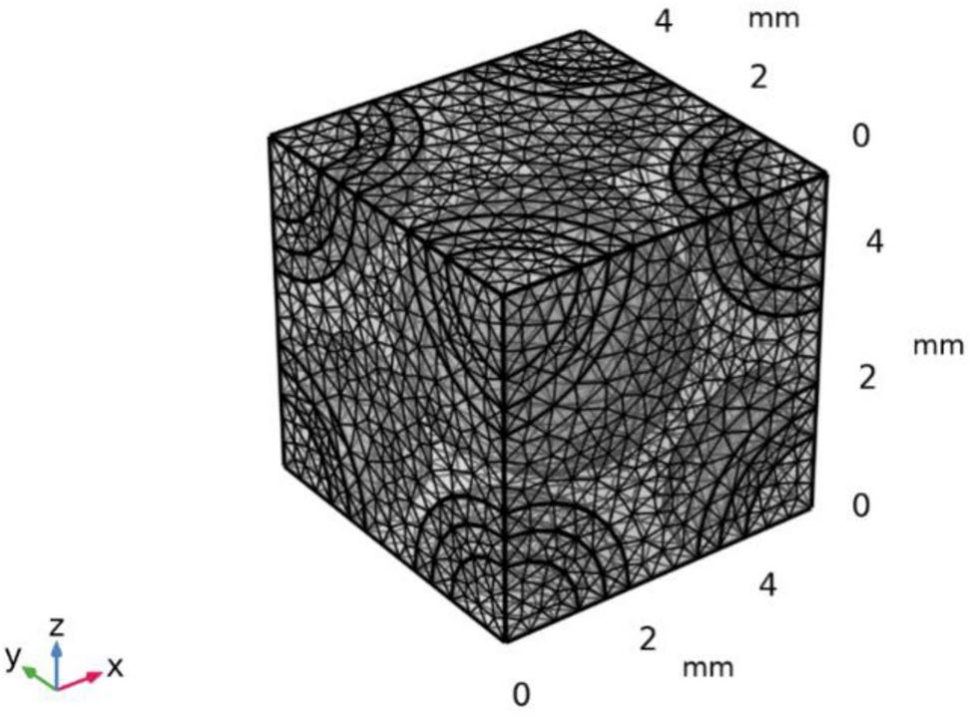
Meshing of a representative volume of green CBC.

For example, Fig. 3 shows a representative volume element of green CBC having the mix ratio of wood dust, cement, fine aggregate and coarse aggregate 0.1: 0.9: 1.5: 3 by volume. Figure 3a–d shows the cubes of 5 mm^3^ highlighting different zone of coarse aggregate, fine aggregate, cement, and wood powder. The solid mechanics module of COMSOL was used for the constitution of the numerical modeling. The physics controlled meshing with fine mesh size (0.05 minimum size) was selected for the study. The mesh consists of 31,012 domain elements, 6456 boundary elements, and 840 edge elements (Fig. 4). The material properties used for modeling are given in Table 1^58^. The linear elastic material model was selected for the study. For a linear elastic material, Hooke’s law relates the stress tensor to the elastic strain tensor:

$$ \sigma = \sigma_{ex} + C:\varepsilon_{{\begin{array}{*{20}c} {el} \\ \\ \end{array} }} $$

$$ \sigma = \sigma_{ex} + C:\left( {\varepsilon - \varepsilon_{inel} } \right) $$

where C is the 4th order elasticity tensor, double-dot denotes tensor product (or double contraction). The elastic strain ε_el_ is the difference between the total strain ε and all inelastic strains ε_inel_.

**Table 1:** Material properties used for modeling^57^.

Material Name/Properties	Young modulus (GPa)	Density (Kg/m^3^)	Poisson’s ratio
Cement	21.9	2050.3	0.279
Wood powder	13	800	0.29
Fine aggregate	26.59	1472	0.24
Coarse aggregate	23.49	1434	0.13

After meshing, the average failure load obtained during the experimentation was applied to concrete and green CBC. The loading and boundary conditions were taken from the experiments during the simulation. The stresses and deformations were obtained for different constituents, interfaces, and zones of the representative volumes of concrete and green CBC.

## Results and discussion:

### Compression testing

**Figure 5:**
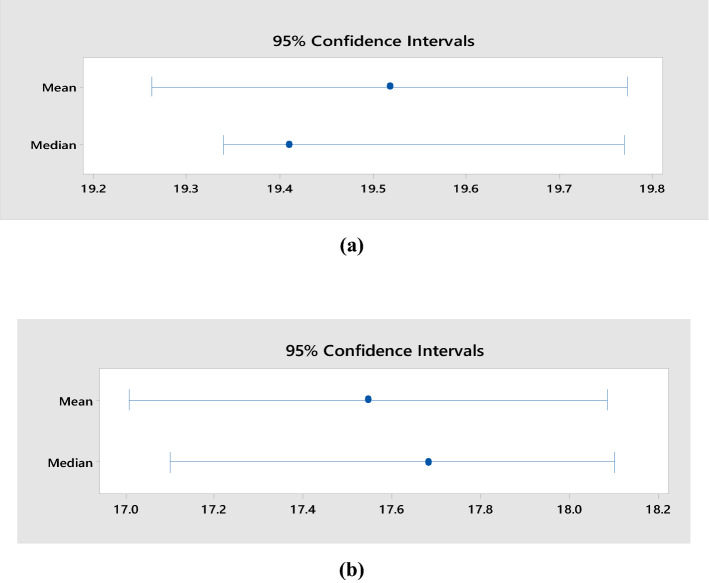
Mean, Median, and variability for compression tests on (**a**) Concrete (**b**) green CBC.

Figure 5a and b show the statistical summary of the compression testing of concrete and green CBC, respectively.

**Figure 6:**
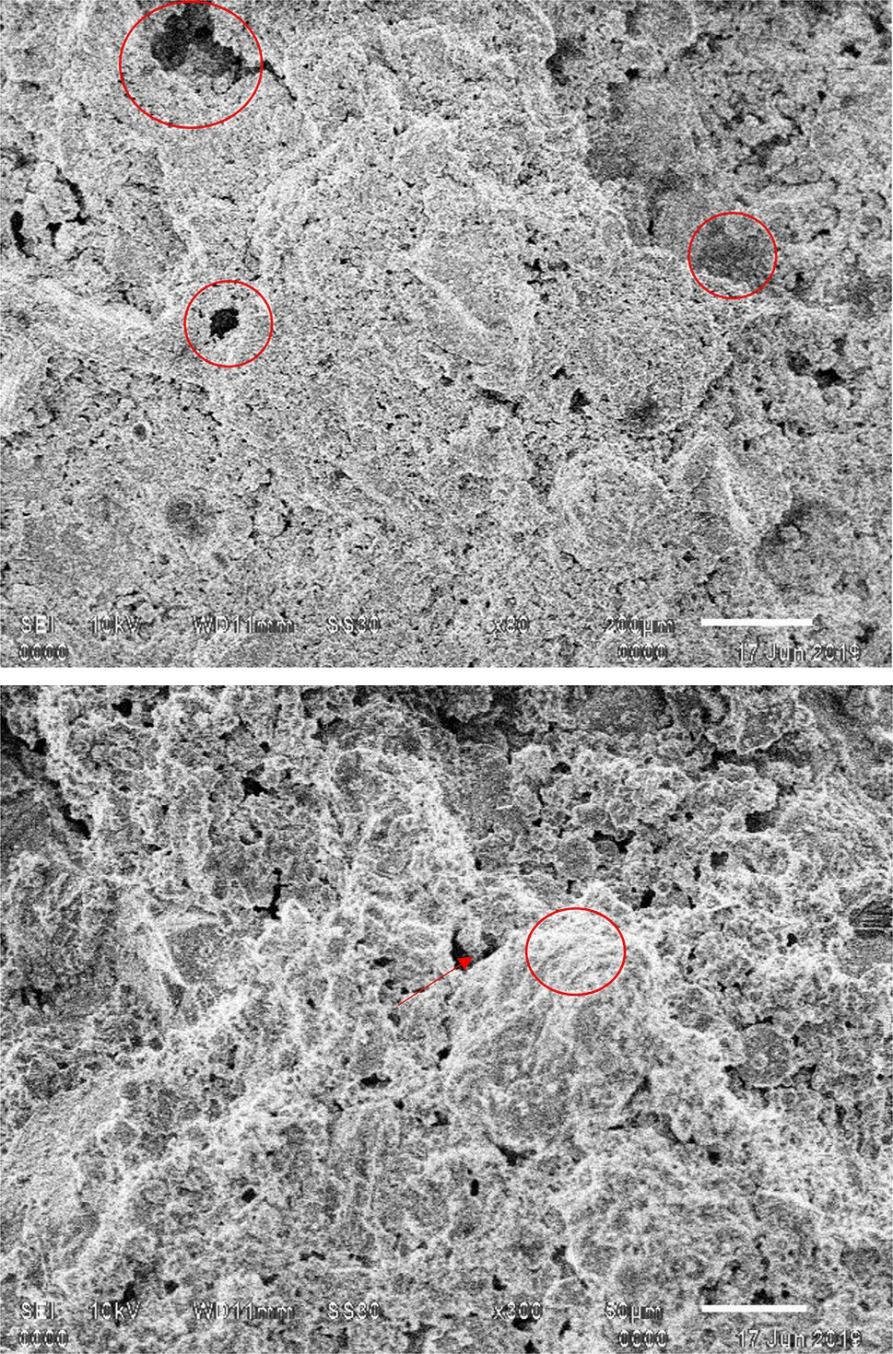
SEM Micrographs of green CBC samples.

The average compressive strength of concrete was 19.51 MPa, whereas for green CBC it was 17.10 MPa. The p-value for concrete and green CBC is higher than 0.05. Thus, these values of compressive strength may not represent the population, but one can safely conclude that the compressive strength degrades due to the addition of wood powder as a replacement to OPC. It was noticed by visual inspection during compression testing that patterns of crack initiation and propagation had two notable differences. First, the edges of the upper side of the green CBC cubes were partially damaged early during the test. Second, the crack propagation in green composites was quicker, and in a few cases, multiple cracks were produced and propagated in various directions simultaneously. The early broken edges of green CBC should be due to stress concentration at the edges, higher stresses due to contact with the loading plate, and insufficient binding properties of wood dust with other constituents. The verification CBC samples had broken edges between 8 and 11.2 MPa for all three samples. The SEM micrographs of fractured green CBC samples shown in Fig. 6 support this theory. It is reflected from the micrographs that the wood particles were having inadequate bonding as the C–S–H gel has not massively spread on the hydrated cement paste. Whereas the concrete samples exhibited adequate binding properties of the OPC paste. The OPC produces better mechanical interlocking at the interface and should be the reason behind the enhanced compressive strength of concrete samples. Additionally, the formation of Portlandite and Calcite over the exterior surface of hydrated cement paste enables the expansion and distribution of mineral elements result in superior compressive strength of concrete. The micrographs of green CBC samples display brittle fractures demonstrated by the speedy propagation of cracks in multiple directions. This should be due to multiple micro-voids and agglomeration of the wood dust particles in the green CBC samples as visible in the micrographs.

### Crack detection using modified spectral clustering

**Figure 7:**
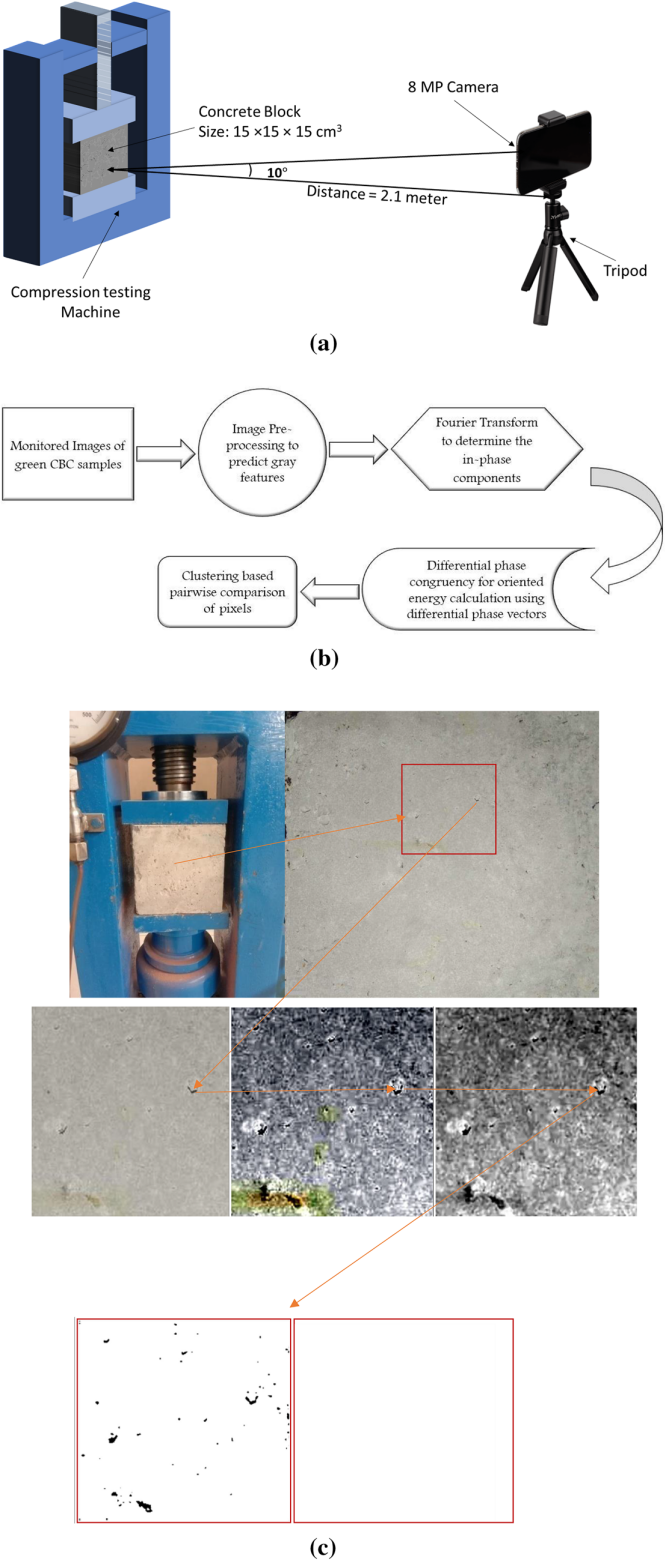
(**a**) Schematic diagram showing experimental arrangement. (**b**) Flowchart showing broad steps of spectral clustering algorithm for crack detection. (**c**) Steps followed by image processing algorithm and removal of noise.

The spectral clustering reduces complex multidimensional datasets into clusters of similar data in odder dimensions. The capability of the developed algorithm for detecting cracks was demonstrated by analysing photographs of the samples during compression testing. More than 500 images are used for crack clustering test. The first photograph considered for the study was the concrete cube sample fitted in the CTM (Fig. 7a, b) at no load. The mobile camera was positioned in front of the compressive machine parallel to the location of clamped sample. Figure 7a shows the schematic including camera positioning and the concrete sample fixed in compression machine. Figure 7b shows the flowchart including broad steps of spectral clustering algorithm for crack detection. The algorithm requires multiple images of the structure featuring surfaces with cracks/ without cracks as input for building the data base. Further, the developed algorithms analyze photographs of the samples and process it to detect any crack present in the surface. The different steps while processing the photograph include the localisation for crack examination followed by the crack segmentation, contrast stretched imaging, gray scaled imaging which produces noisy spectral clustered image and finally the image with crack detection, if any, after removal of noise by morphological examination. The original work based on spectral clustering works on eigenvalues and eigenvectors of the similarity matrix in target and left many unresolved problems associated with it. The traditional spectral clustering is sensitive to the initial conditions and multiple starts as it uses k-means clustering at the root. Besides using the initial conditions, we have utilized the dividing of the data based on signs such as positive and negative values. Also the Laplacian matrix has been tested under slight change in the diagonal parameters of the diagonal matrix as diagonal parameter gives the highest similarity information to form a strongly connected cluster of the edge pixels.

**Figure 8:**
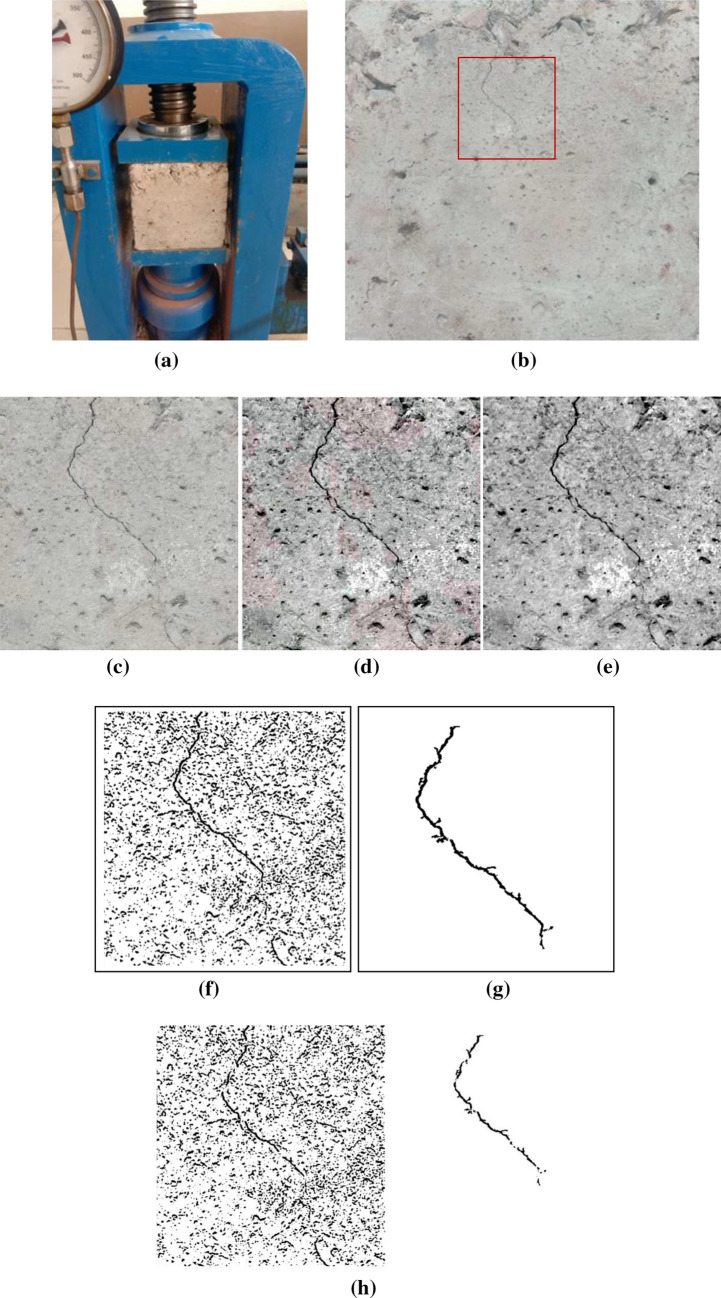
(**a**) Compression testing setup with CBC having minor crack (**b**) Small crack developed and localised (**c**) Crack segment (**d**) Contrast stretched image (**e**) Gray scaled image (**f**) Noisy spectral clustered image (**g**) Noise-free (**h**) Crack detection with spectral clustering method without modification.

**Figure 9:**
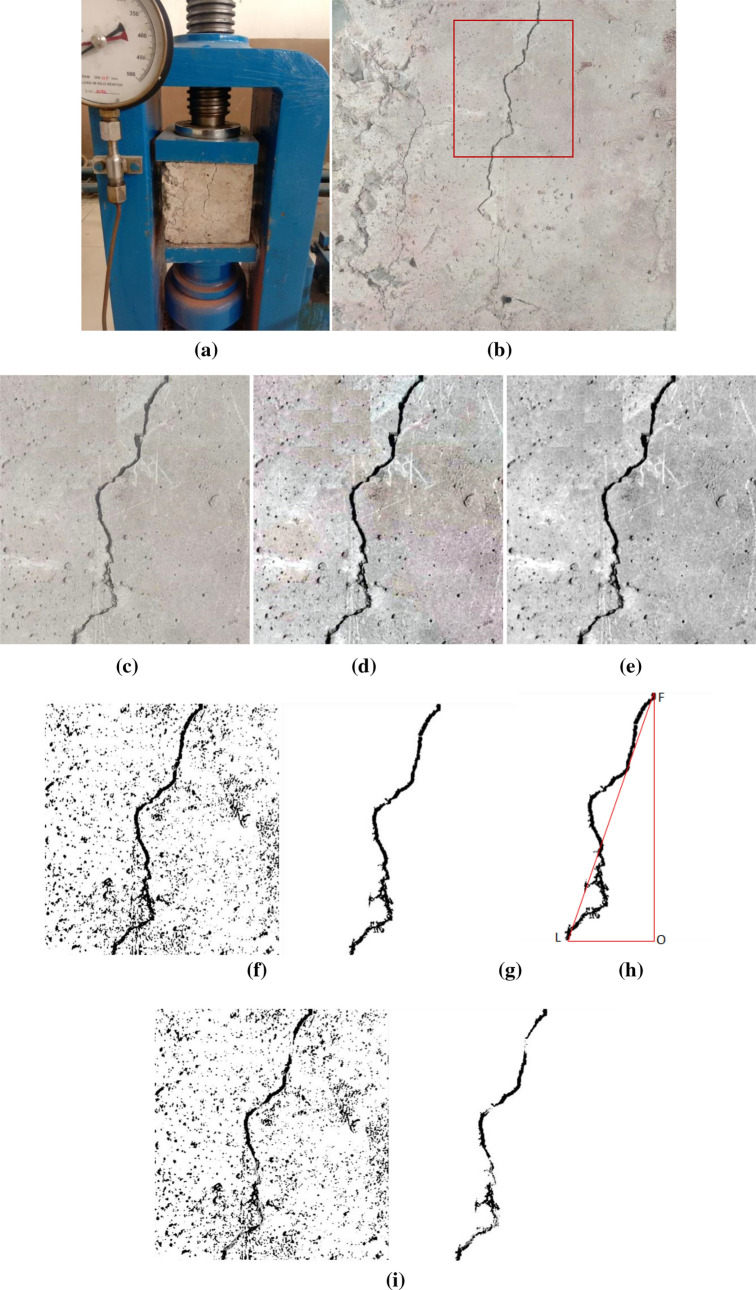
(**a**) Compression testing setup of CBC with major crack (b) Major crack developed and localised (**c**) Crack segment (**d**) Contrast stretched image (**e**) Gray scaled image (**f**) Noisy spectral clustered image (**g**) Noise-free crack (**h**) crack length measurement (**i**) Crack detection with spectral clustering method without modification.

Figures 7c, 8 and 9 shows the results of the block-wise segmented images. The input images are processed first by dividing into multiple parts of non-overlapping blocks of fixed size 32 × 32 through spectral clustering. The spectral clustering analysis performed for shows that the algorithm has removed the noise present successfully in the sample photograph and not detected any crack.

Figures 8a and 9a shows the sample photographs reporting the crack initiation and propagated crack, respectively. Figures 8d, e and 9d, e are the pre-processed image for the image contrast stretching and red, green, blue (RGB) to gray conversion. The noisy spectral clustered images of the crack are shown in Figs. 8f, 9f. The noise-free images have been shown in Figs. 8g and 9g for the smaller and larger crack, respectively. In the present work, there was no requirement of separate preprocessing to filter the unwanted noise from the image before the feature extraction. For reducing the sensitivity to noise present in the image, a differential phase congruency was utilised instead of phase congruency. Inclusion of differential phase has improved the accuracy of the spectral clustering and eliminated the effects of noise present in the image.

We have implemented the traditional spectral clustering algorithm for detection of crack in the same image and compared the results with modified algorithm. As compared to the simple phase congruency in traditional spectral clustering, the crack detected using modified spectral analysis with differential phase congruency based spectral clustering and previously stated improvements are much better as reflected by the Fig. 8h and 9i.

### Crack length measurement

The crack length was measured by measuring the distance between the farthest pixel either in vertical or horizontal direction with certain angle. The end-point distance between pixel has been measured in present work by the following method:

*Step 1* Detect the crack through spectral clustering.

*Step 2* Start from with the pixel f(0,0) of the crack image to determine the first pixel in the row and column through iterative process for rows and columns until first pixel found.

*Step 3* Find the starting point of the crack by comparing the adjacent pixel in horizontal and vertical direction to predict the crack propagation.

*Step 4* Find the last pixel through iterative process of the crack depending upon the propagation of the crack either in vertical or horizontal direction.

*Step 5* Now determine the longest geometrical distance between first and last pixel by considering the :

$$ {\text{length}} = \left( {{\text{OF}}_{{{\text{vertical}}}}^{2} + {\text{OL}}_{{{\text{horizontal}}}}^{2} } \right)^{{{\raise0.7ex\hbox{$1$} \!\mathord{\left/ {\vphantom {1 2}}\right.\kern-\nulldelimiterspace} \!\lower0.7ex\hbox{$2$}}}} $$

where OF is the distance perpendicular from the first pixel (F) to the line of the last pixel (at pixel O) and OL is the linear distance in direction till the last pixel (pixel L) from the point receiving the perpendicular(at pixel O) from pixel F.

Finally, the length of the crack is calculated and calibrated in mm through number pixel converted in to equivalent crack length. In Fig. 9h 652 pixles lengths, transform into actual length is 38.06 mm. For the image quality available in the present work, the crack of < 5 mm size can be detected easily.

### Finite element model

**Figure 10:**
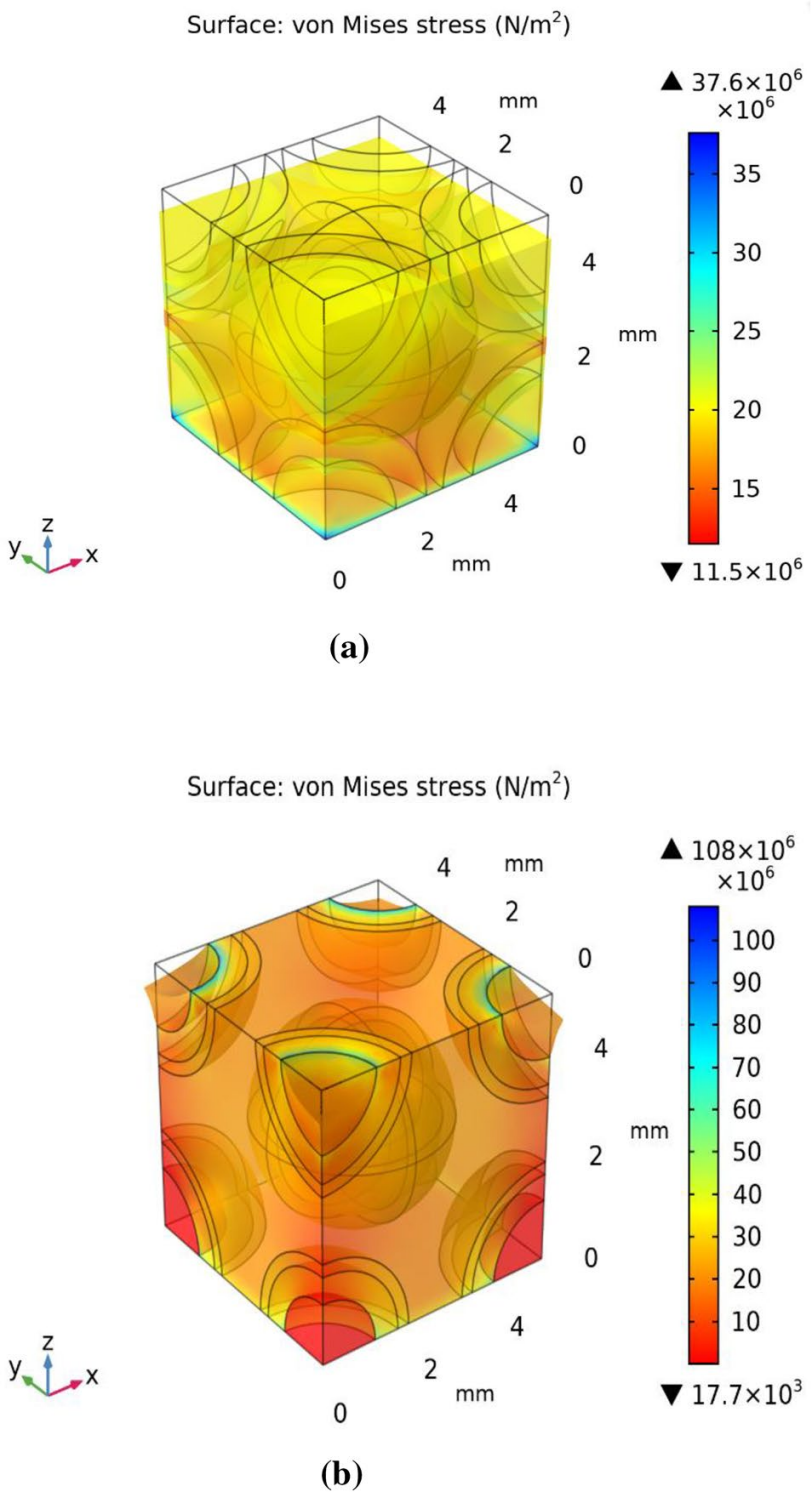
Von Mises stress after application of 20 MPa (**a**) Concrete (**b**) Green CBC representative volume.

**Figure 11:**
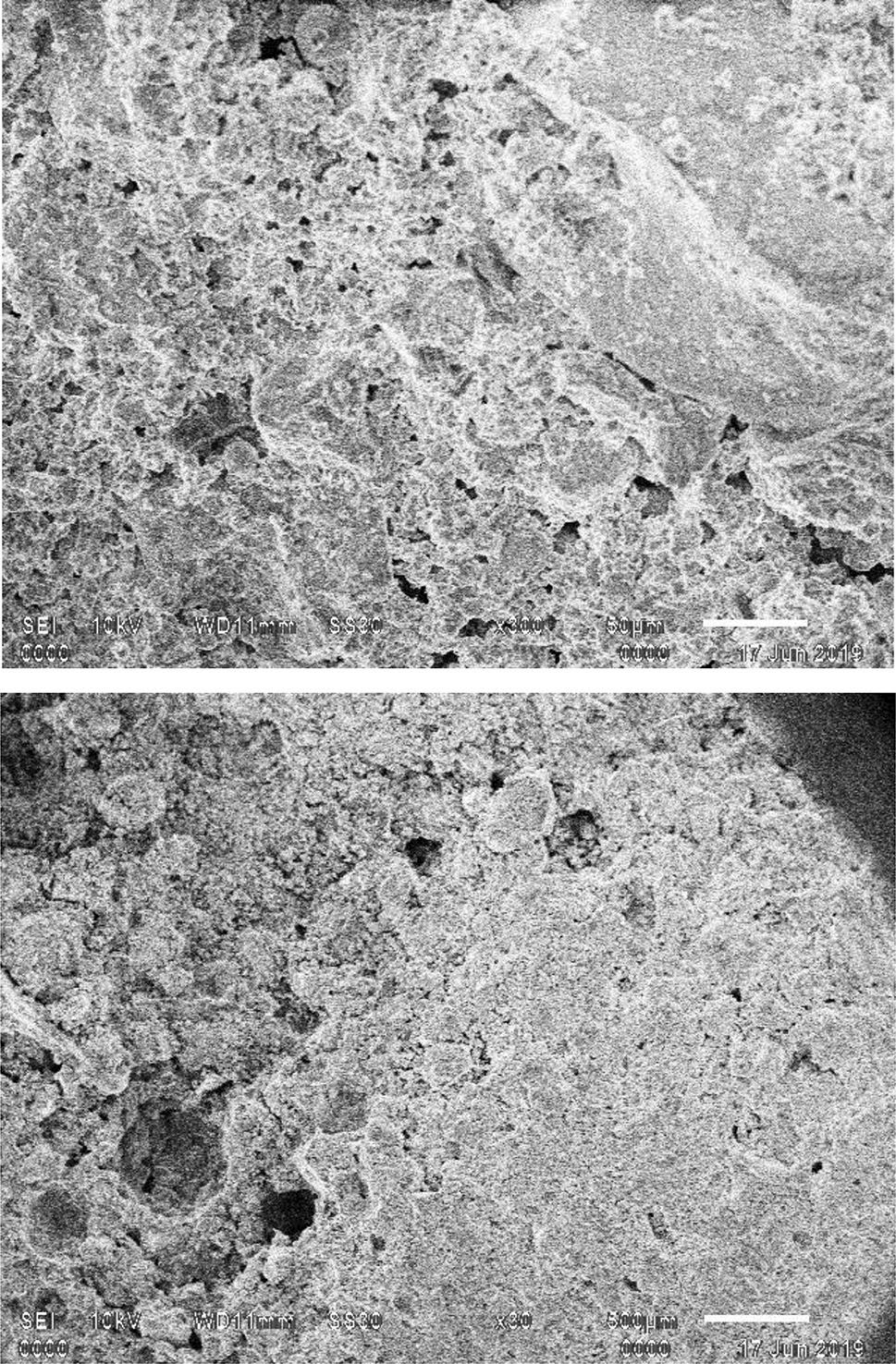
SEM micrograph of the fractured verification CBC samples (Corner) with higher wood content at the corners.

The FE model employed in this study was derived from the unit-cell approach and the volume proportion of the each component of cement based composite in model follows the real constituent proportion. Figure 10a and b shows the distribution of Von Mises stress in the different constituents and their interphases of Concrete and Green CBC representative volumes respectively after application of 20 MPa load. The results of the FE model clearly represent the zones prone to fracture first due to the higher stresses. It is clearly visible that the stresses are highest at the interfaces of wood powder. The stresses vary from 11.5 to 37.6 MPa and 17.7 to 108 MPa for concrete and CBC respectively. A considerably high maximum stress is reported in CBC than in concrete for the same loading. It is visible in Fig. 10a that stresses and deformation in concrete are of uniform in nature, slightly higher stresses in the upper half zone than the bottom half zone while the stresses in CBC are non-uniform nature (Fig. 10b) with comparison to concrete. The stresses at the bottom corners of CBC are less i.e. near to 17.7 MPa while the stresses in wood dust zone at the upper corner of CBC are the highest. The deformation is the highest at the top edges of CBC. The upper corners are deformed out of the cube that leads to crack. The fracture should have begun from the interfaces of wood dust with other constituents. It could be now safely predicted that the corners of the green CBC sample must have broken due to the presence of wood dust. This theory was verified with an additional experimental run with the specially prepared samples having higher wood dust content at the corners. This verification experiment has ensured the correctness of the model results. Figure 11 shows the SEM micrographs of verification CBC samples. The sample for SEM was prepared from the fractured edges. The presence of wood dust in a more significant amount is visible. SEM micrograph shows that the green CBC is comparatively heterogeneous to concrete. However, C-S–H was less developed because of unreacted particles. The dilution, agglomeration, and filler effect have resulted in lower compressive strength of green CBC^59^. The dilution due to partially replacing OPC with the wood dust leads to fewer hydration products and a weaker microstructure. Additionally, the wood dust agglomeration created a fragile zone in the microstructure, and lack of hydration of particles could take place in the hardened cement matrix.

**Figure 12:**
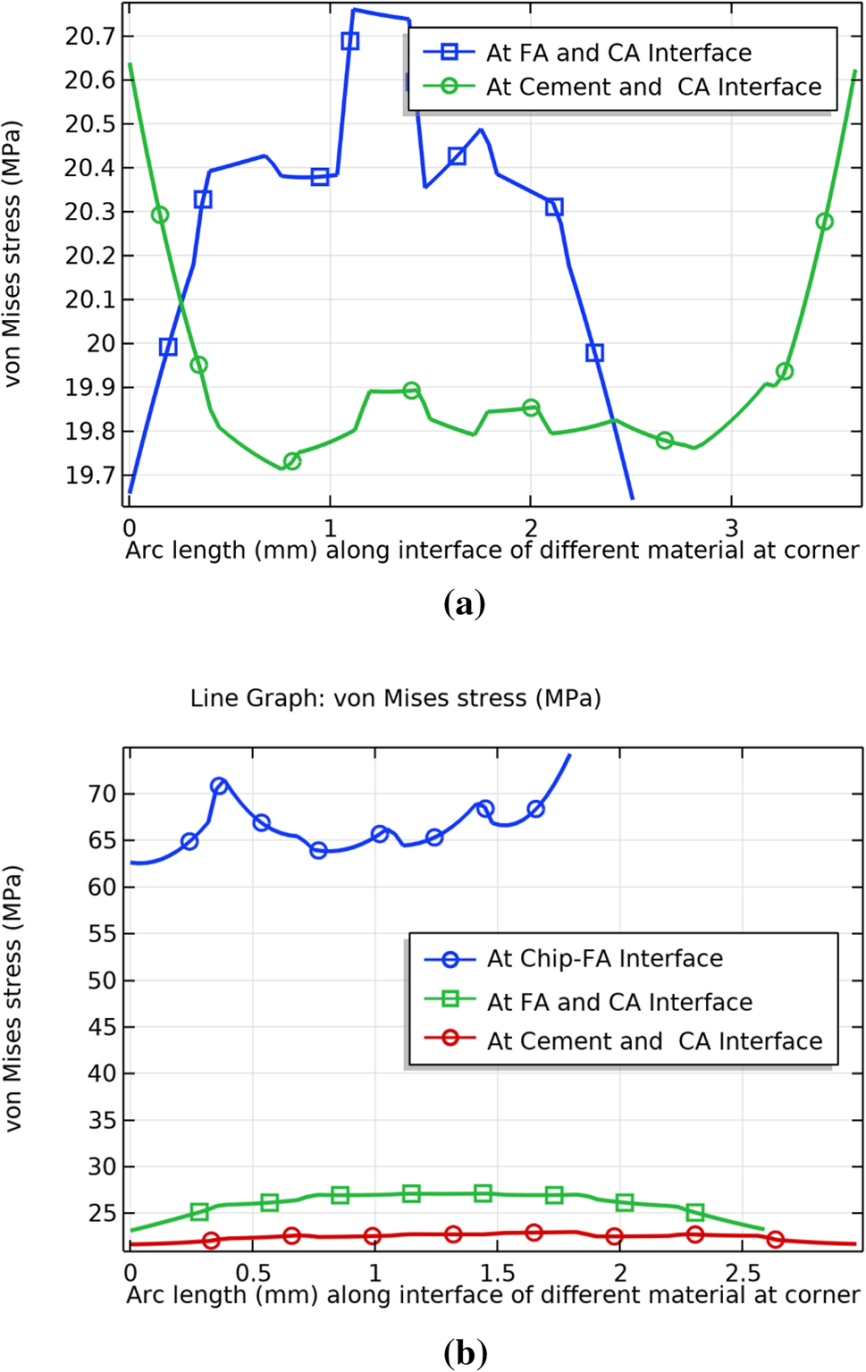
Von Mises stress at the interfaces of (**a**) Concrete (**b**) Green CBC representative volume.

The SEM micrographs (Fig. 11) show that uniform distribution is not commonly observed in composites. However, literature frequently reports use of representative volume elements (RVE) for analysing such composites effectively^57,60–64^. The stress distribution can be effectively predicted using this approach in variety of composites including cement-based composites. The stresses at the interface of coarse aggregate (CA), fine aggregate (FA), cement, and wood dust were studied. Figure 12 reflects the line graphs showing stresses on the upper plane of the concrete and CBC samples Fig. 12a and b reflect that at wood dust and fine aggregate interfaces have considerably high stresses in comparison to the other interfaces. This highlights the possibility of crack initiation from the wood dust-FA interface. However, the stresses at the interface near to the center of the CBC is decreasing as shown in Fig. 12b.

**Figure 13:**
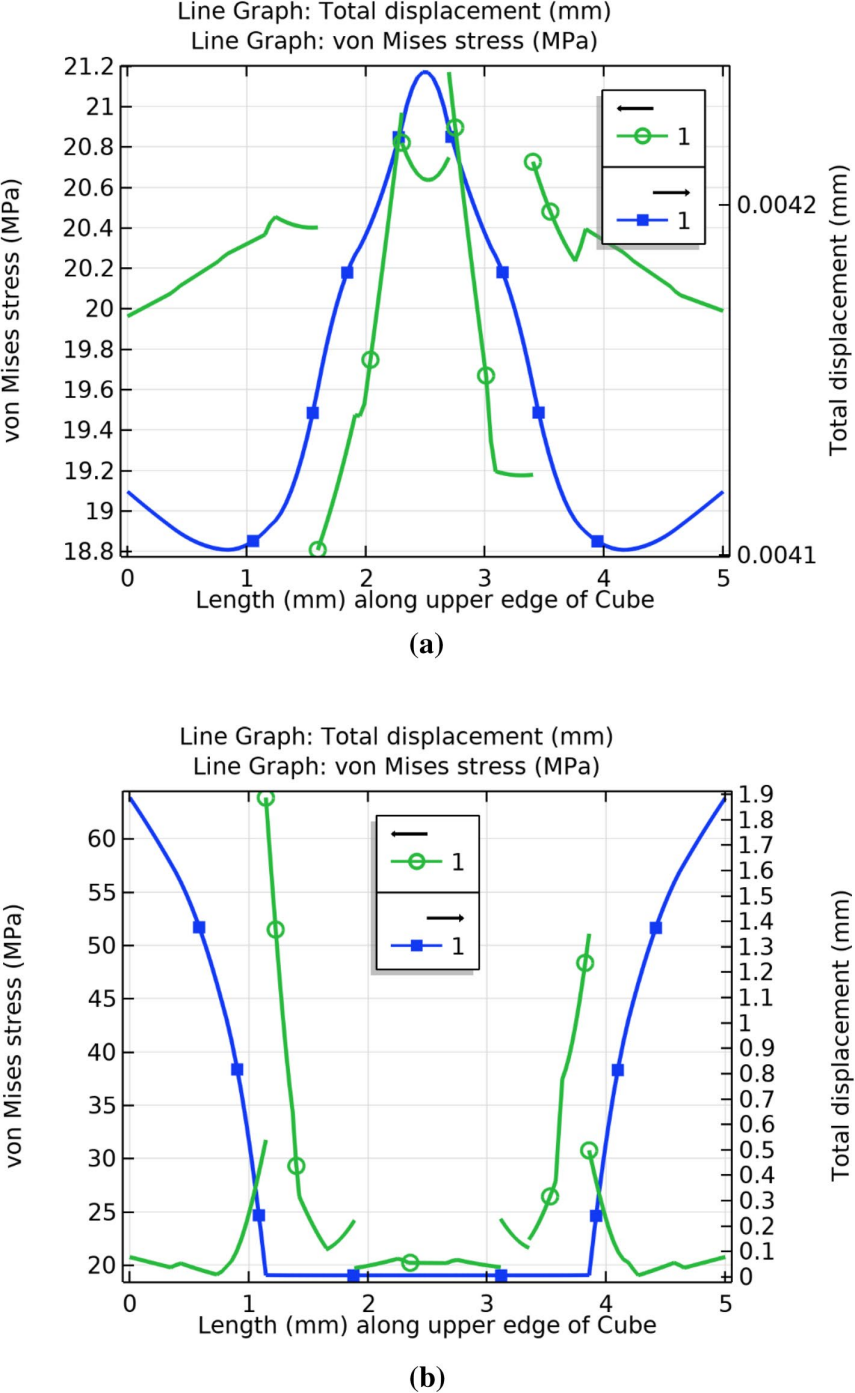
Von-Mises stress and total displacement along the upper edge of (**a**) Concrete (**b**) Green CBC representative volume.

**Figure 14:**
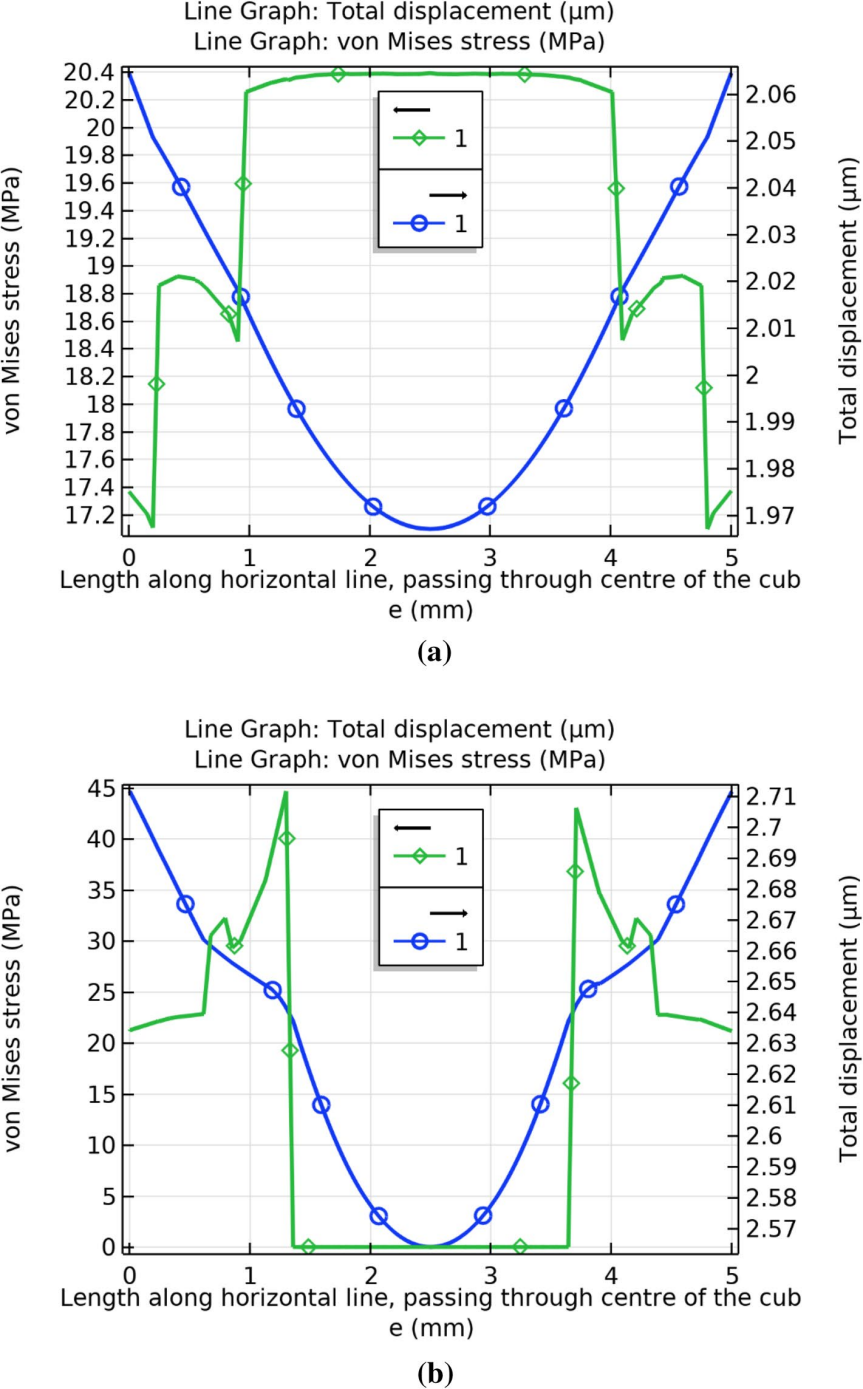
Von Mises stress and total displacement along center of (**a**) Concrete (**b**) Green CBC representative volume.

In order to support the theory that the FA-wood dust interface contributes to the fracture initiation, the difference between the stresses and displacement of concrete and green CBC was investigated. Figure 13 clearly shows that the Von Mises stresses and total displacement along the upper edge of green CBC are considerably higher than the concrete. Thus, it is apparent that the broken edges of the green CBC are due to the high displacement resulted because of the high stresses of the FA wood dust interface. The crack should not boundaries from the center of the concrete or green CBC material under compressive loading as stresses and displacement are lower at the center and the nearby region, as reflected by Fig. 14. Figure 14a shows that the stresses at the center of cube is maximum and decreases sharply at the interface of different materials and deformation is minimum at the center of concrete while Fig. 14b shows the stresses is at the center of cube is minimum and increases sharply at the interface of different materials and deformation is also minimum at the center of green CBC. Therefore, it can be concluded that in case of impurity/void present or degradation due to aging there may be a possibility of crack from the centre in the concrete, but the crack possibility from the centre should be minimum in green CBC under compressive loading.

Thus, the FE model and morphology examination could be used for the identification of crack prone zone in green CBC structure if properties of constituents are known and loading conditions could be defined appropriately. The images of the crack prone region if the input to developed algorithm information related to the severity of crack or requirement of maintenance could be obtained without the necessity of skilled staff for inspection.

## Conclusion

The present work proposes an initial investigation for a methodology that will provide inputs for unmanned structure maintenance using a modified spectral clustering algorithm, FEM, and SEM investigation. The methodology was applied on conventional concrete and green composite based composites (CBCs) incorporating saw dust subjected to compressive strength tests. The main findings of the study were:

- The developed image processing algorithm can effectively extract the crack features from the concrete crack images taken with an ordinary mobile phone camera. Some pre-processing steps are required for proving useful input to the spectral clustering algorithm.
- Cracks were detected by the dissimilarity between the surface energy of the pixel pairs. The algorithm is capable of overcoming the environmental noises, and any other noise appeared during image analysis.
- Damage-prone zones can be predicted effectively using the developed numerical model.
- The FE model and image processing collectively develop a framework for crack detection at an early age with continuous automated observation. The smaller, larger and multiple cracks in concrete and CBCs can be captured and measured in real-time. Crack length can be measured through standard function in the image toolbox of the MATLAB.
- The replacement of OPC with wood dust reduced compression strength and produced different fracture pattern regarding reference concrete. Conventional concrete samples showed compressive strength 10.25% higher than green CBC samples.
- The lower strength of green CBC and crack prone zone was determined through FE method and matched with SEM micrographs of fractured CBC samples.
- The method allows timely intimation about the damage in structure for appropriate action through images from inspected zones without requiring any expert from the same domain. The implementation of developed methods for real time structural health monitoring requires additional efforts and considered as a future work.
